# Genotyping and Characterizing *Plasmodium falciparum* to Reveal Genetic Diversity and Multiplicity of Infection by Merozoite Surface Proteins 1 and 2 (*msp-1* and *msp-2*) and Glutamate-Rich Protein (*glurp*) Genes

**DOI:** 10.3390/tropicalmed9110284

**Published:** 2024-11-20

**Authors:** Muharib Alruwaili, Abozer Y. Elderdery, Hasan Ejaz, Aisha Farhana, Muhammad Atif, Hayfa Almutary, Jeremy Mills

**Affiliations:** 1 Department of Clinical Laboratory Science, College of Applied Medical Sciences, Jouf University, Sakaka 72388, Saudi Arabia; hetariq@ju.edu.sa (H.E.); afarhana@ju.edu.sa (A.F.); maahmad@ju.edu.sa (M.A.); 2 Medical Surgical Nursing Department, Faculty of Nursing, King Abdulaziz University, Jeddah 21589, Saudi Arabia; aalalmetere2@kau.edu.sa; 3 School of Medicine, Pharmacy and Biomedical Sciences, University of Portsmouth, Portsmouth PO1 2DT, UK; jeremy.mills@port.ac.uk

**Keywords:** *Plasmodium falciparum*, genetic diversity, molecular markers, multiplicity of infection

## Abstract

Resistance to current antimalarial drugs is steadily increasing, and new drugs are required. Drug efficacy trials remain the gold standard to assess the effectiveness of a given drug. The World Health Organization (WHO)’s recommendation for the optimal duration of follow-up for assessing antimalarial efficacy is a minimum of 28 days. However, assessing antimalarial drug efficacy in highly endemic regions can be challenging due to the potential risks of acquiring a new infection in the follow-up period, and thus, it may underestimate the efficacy of the given drugs. A new treatment should be introduced if treatment failure rates exceed 10%. Overestimation occurs as a result of retaining a drug with a clinical efficacy of less than 90% with increases in morbidity and mortality, while underestimation may occur due to a misclassification of new infections as treatment failures with tremendous clinical and economic implications. Therefore, molecular genotyping is necessary to distinguish true new infections from treatment failures to ensure accuracy in determining antimalarial efficacy. There are three genetic markers that are commonly used in antimalarial efficiency trials to discriminate between treatment failures and new infections. These include merozoite surface protein 1 (*msp-1*), merozoite surface protein 2 (*msp-2*), and glutamate-rich protein (*glurp*). The genotyping of *P. falciparum* by nested polymerase chain reaction (n-PCR) targeting these markers is discussed with the inherent limitations and uncertainties associated with the PCR technique and limitations enforced by the parasite’s biology itself.

## 1. Introduction

*Plasmodium falciparum* is the most common and harmful species of malarial parasite that infects humans [1]. There were approximately 241 million documented cases and 627,000 fatalities attributed to malaria worldwide in 2023 [2]. Africa is the home of more than 95% of malarial infections and deaths [2]. Resistance towards antimalarial drugs and the emergence of resistant mosquitoes to insecticides are the main hurdles to the control and elimination of malaria [3].

Resistance to *P. falciparum* is a growing concern globally since resistance to almost all available antimalarial drugs has been reported [4]. Resistance is defined as the “capacity of a strain of parasite to persist and reproduce even when a medicine is given in dosages that are equivalent to or greater than the typically advised amounts, but the subject is still able to tolerate it” [5]. Recurrent or persistent *P. falciparum* infection up to 28 days after treatment with antimalarial drugs is a sign of resistant parasites [6]. Hence, it is imperative to expedite the development of novel antimalarial therapies and closely observe the emergence and spread of resistance parasites [7].

The mechanisms underlying genetic resistance towards antimalarial drugs have been demonstrated for certain drugs [8]. Single nucleotide polymorphisms (SNPs) in the *P. falciparum* dihydrofolate reductase gene (*pfdhfr*) provide resistance to pyrimethamine, whereas mutations in the *P. falciparum* dihydropteroate synthase gene (*pfdhps*) mediate resistance to sulfadoxine [9,10]. In addition, chloroquine resistance has been attributed to a key gene called the *P. falciparum* chloroquine resistance transporter gene (*pfcrt*), which is also linked to decreased sensitivity to amodiaquine [11]. Other genes have also been identified, such as *P. falciparum* multidrug resistance protein 1 (*pfmdr1*) and multidrug resistance-associated protein (*pfmrp1*), which encode proteins located on the digestive vacuole or the plasma membrane [12].

In antimalarial drug efficacy studies, resistance markers would not be sufficient to discriminate between recrudescent and new infections. Even though in vitro assays are standard methods to determine resistance, they can be confusing as not all parasite strains grow equally. In addition, sequestered parasites in deep tissues may go undetected during sampling [13,14].

For practical purpose, resistance to a given antimalarial drug can be estimated by comparing the treatment failures to the number of successfully treated individuals [5,6]. However, treatment failures might occur due to recrudescence or new infections [5]. Recrudescence is defined as the recurrence of the asexual stage of parasites of the same genotypes that caused the initial infection [5]. New infections result from new inoculation by mosquitoes, resulting in the introduction of new parasites which are more likely to be genetically distinct. Given the fact that the incubation period of *P. falciparum* might be as brief as seven days, in areas with high malaria transmission, new infections cannot be ruled out, and therefore, it is difficult to discriminate recrudescence from new infections, which may influence the accuracy of resistance estimates to a given drug [15]. Most antimalarial drugs act on asexual erythrocytic blood stages; parasites like *P. vivax* and *P. ovale* develop hypnozoite stages, resulting in a relapsing form classified as recrudescence [8].

In antimalarial drug efficacy studies, there is a need for a technique to differentiate between recrudescence and new infections to better estimate the efficacy of a given drug [16]. *P. falciparum* is known to be genetically diverse, and this genetic diversity has been studied intensively with respect to many aspects [17,18,19]. The simplest polymorphisms found in *P. falciparum* are SNPs resulting from a single base substitution, and most of these mutations are associated with non-synonymous mutations, such as mutations found in *pfdhfr*, *pfdhps*, and *pfcrt*, which confer resistance to antimalarial drugs [9,10,11]. Other polymorphisms are attributed to the presence of repetitive sequences, and based on their number and arrangement, parasite lines can be classified into distinct families [20,21]. While any polymorphic sequences can be utilized as genetic markers, there are some conditions which must be met in order to consider a specific sequence suitable as a genetic marker. These conditions include the following: (1) a given sequence must present in a single copy, (2) the polymorphic region has to be found in all of the parasites, and no null variants can be found naturally, and (3) the variable region needs to be stable and cannot mutate through the successive mitotic division during asexual reproduction. The effectiveness of genetic markers depends on the degree of polymorphism, the frequency of these markers in a specific population, and the ease with which these genetic markers can be detected and discriminated.

In *P. falciparum*, three genes have been widely used as genetic markers, and these genes include *msp-1*, *msp-2*, and *glurp* [22,23,24] (see Table 1). These genes are considered the most commonly selected genetic markers due to the fact that these loci are situated on different chromosomes, which decreases the likelihood of linkage, and because they are highly polymorphic [20,25]. In this review article, we discuss the importance of these genes in characterizing the genetic diversity and multiplicity of infection (MOI) of *P. falciparum*.

## 2. The *msp-1* Gene

**Figure 1:**
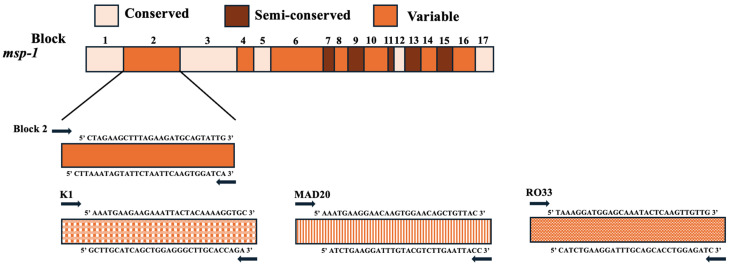
Diagrammatic representation of *msp-1* gene of *P. falciparum*.

The *msp-1* gene is situated on chromosome 9 and encodes a surface protein on the merozoite with a molecular weight ranging from 190 to 200 kDa [26,27]. Merozoite is the infecting stage to erythrocytes, and therefore, *msp-1* is the basis for one of the vaccine candidates [28]. Even though the function of *msp-1* is not fully understood, it has been suggested that it is essential for *Plasmodium* development [29]. In addition, it has been thought that *msp-1* plays a significant part in the attachment and invasion of erythrocytes [30,31]. *msp*-1 can be segmented into 17 blocks, each flanked by conserved areas, based on the arrangement of its amino acids [26,27]. Block 2 exhibits the highest degree of polymorphism and contains a highly repetitive variable region through which, based on the number of repeats and their arrangement, *msp-1* can be classified into three allelic families: MAD20, K1, and RO33 [32,33]. Block 2 encodes 65 amino acids and is predicated to be unstructured [34]. The schematic gene structure and primers used to amplify conserved and polymorphic regions are shown in Figure 1.

Block 2 is the most polymorphic region and comprises three allelic families: K1, MAD20, and RO33. The primer sequence used to amplify this region can be found in Table 2.

## 3. The *msp-2* Gene

**Figure 2:**
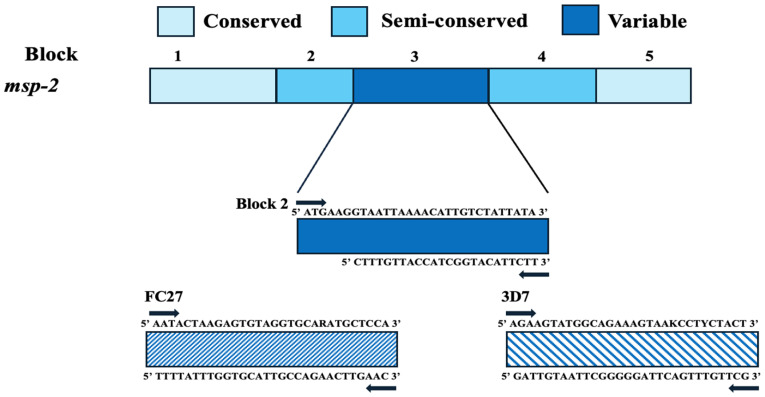
Diagrammatic representation of *msp-2* gene of *P. falciparum*.

The second most prevalent merozoite surface protein is *msp-2*. This protein is encoded by the *msp-2* gene, which is situated on chromosome 2 and has a molecular weight of 30 kDa [23,32]. Like *msp-1*, *msp-2* seems to be involved in invasion; its the exact role remains unclear, but specific antibodies against *msp-2* have been associated with some sort of protection in endemic regions [35,36]. The *msp-2* gene consists of five domains, with the central domain being a highly polymorphic region. Although multiple alleles can be found and detected, *msp-2* is divided into two major allelic families known as FC27 and 3D7 [23]. A schematic diagram of *msp-2* is illustrated in Figure 2.

Block 3 is the most polymorphic region and comprises two allelic families, FC27 and 3D7. The primer sequence used to amplify this region can be found in Table 2.

## 4. The *glurp* Gene

**Figure 3:**
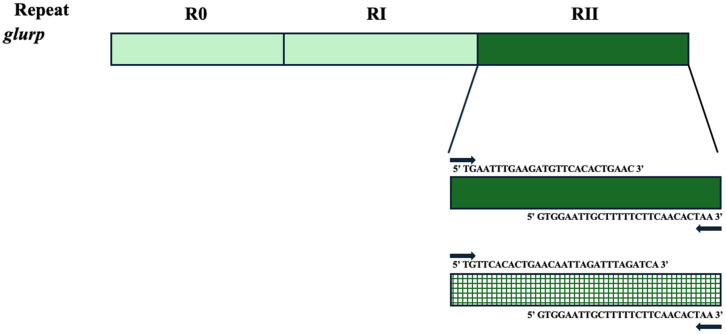
Diagrammatic representation of *glurp* gene of *P. falciparum*.

The *glurp* gene is one of the polymorphic antigens which can act as a genetic marker to characterize *P. falciparum* populations [20]. The gene is situated on chromosome 10 and codes for a 220 kDa protein which is expressed in all phases of the parasite’s asexual reproduction [24,37]. The *glurp* is localized on the merozoite surface in a complex protein called Ps38 [38]. This complex protein binds to the erythrocyte through glycoprotein A and acts as a receptor [38]. Evidence supports the function of this gene as an immune target, suggesting that an immune response against this gene may confer some protection [39]. The *glurp* gene comprises three defined areas, namely R0 (N-terminal non-repetitive), R1 (central repetitive sequence), and R2 (C-terminal repetitive domain) [37]. In fact, antibody levels against the R0 and R2 domains were positively correlated with a decreased risk of symptomatic malaria [40]. Despite the fact that the R2 region is conservative compared to other regions, the number of repeat units of amino is variable amongst *P. falciparum* isolates, leading to a size polymorphism [41]. Therefore, the *glurp* gene is one of the genetic markers commonly used as a genetic tool in many studies to characterize the MOI and genetic diversity in *P. falciparum* populations, indicating the importance of this marker [42,43,44]. The *glurp* schematic structure is demonstrated in Figure 3.

C-terminal repetitive region RII consists of repeat units, and their numbers and arrangements differ between parasite clones. The primer sequence used to amplify this region can be found in Table 2**.**

## 5. Genetic Measurements Used to Characterize Genetic Diversity in *P. falciparum*

When genotyping *P. falciparum* populations using the above genetic markers, numerous genetic measurements like expected heterozygosity (*H_E_*), MOI, and linkage disequilibrium (LD) can be used to estimate the factors influencing *P. falciparum*’s population structure [45,46,47,48]. The first two are the most common measurements since they are easily calculated, and their values are relevant to malaria transmission. The expected heterozygosity measures the loci diversity and is defined as the likelihood that at a given locus, any two alleles are different from each other when chosen randomly from a specific population [49]. It is calculated based on the following formula: [n/n − 1] [1 − Σ*P*^2^]. n represents the sample size, and *P* represents the allele frequency. *H_E_* has a potential value ranging from 0 to 1, where 0 indicates no allelic diversity, while 1 means that all sampled alleles are different [49]. The MOI refers to the mean number of distinctive *P. falciparum* genotypes per infected person. The calculation involves dividing the number of *P. falciparum* genotypes detected by the total number of samples that are positive [50].

The expected heterozygosity and MOIs have been studied in multiple malaria-endemic regions, and they perform a key role in understating malaria transmission [51,52]. In fact, highly malaria-endemic regions tend to be associated with elevated levels of *H_E_* and a high MOI, while in contrast, low genetic diversity and MOI are reported in areas with low malaria transmission settings like South America and Southeast Asia [53,54,55,56]. Intensive malaria control measures contribute to the reduction in malaria spread in many areas in sub-Saharan Africa, suggesting that the population structures of *P. falciparum* in these regions may become similar to populations in regions with a low incidence of malaria. In fact, the intensification of malaria prevention and control measures has shown to reduce malaria transmission and influence the genetic diversity and population structures of *P. falciparum* [57,58,59]. Hence, genetic diversity and MOI can be employed to evaluate the usefulness of malaria control strategies and to monitor the consequences of eradication programs. In addition, genotyping *msp-1*, *msp-2*, and *glurp* may help in tracing parasite clones with time in cohort studies and evaluate the infection’s duration [60,61]. More importantly, these genetic markers are useful in distinguishing between recrudescence and new infections of *P. falciparum* in antimalarial drug trials [16,42].

Discriminatory power of markers: The discriminatory power of a particular marker relies on the amount of diversity of alleles and their frequency in the circulating parasite population [20]. The discriminatory power of *msp-1*, *msp-2*, and *glurp* markers is still debatable. While some research states that *msp-2* is the most powerful with more alleles detected, other studies have reported that *msp-1* is more powerful than *msp-2* [16,44]. Even though the selection and number of genetic markers are still controversial, the recommended strategy for *P. falciparum* genotyping in antimalarial drug efficacy studies stipulate conducting a consecutive analysis starting with a highly polymorphic genetic marker and moving towards the least polymorphic marker [16]. As the number of genetic markers increases, the probability of detecting different alleles from two independent samples also increases [62]. Therefore, identical alleles observed in two samples taken from the same individual might indicate that infection is more likely to be attributable to recrudescence. In such a case in which alleles are distinct, a new infection can be assumed [16]. However, this is especially difficult in highly endemic regions where multiple infections are common.

Techniques to analyze genetic markers: Primary and nested polymerase chain reaction (nPCR) methods targeting an allele-specific family region on the parasite are the most commonly used methods to genotype parasite populations [20]. The advantages of this type of method is that it includes increasing sensitivity to detect minor alleles, and minor variations in amplification conditions means they have little or no impact on detection sensitivity [20]. However, there are of course downsides with this methodology as it is time-consuming and additional materials are required [63]. In addition, there is a substantially increased risk for contamination, which can be mitigated if good lab practice precautions are employed [20]. Several primer sequences have been published in the literature, so a primary PCR is performed by first targeting conserved sequences flanking polymorphic regions, and this is then followed by nested PCR using the product of the first PCR reaction as the DNA template to amplify the polymorphic regions. The primers’ names, sequences, and targeted regions are illustrated in Table 2 [20]. The conserved sequences and flanking polymorphic areas are distinct to each family and shared among allelic variants within the same family. Depending on the number of families present, pairs of primers can be used to amplify the polymorphic region on each family, and the allelic variant will be detected [20].

PCR products are then analyzed by electrophoresis, for example, as they can be easily visualized even if they are obtained from a single parasite. Identifying allelic variants is achieved by determining the size of the DNA fragment following gel electrophoresis.

Restriction fragment length polymorphism (RFLP) is another genotyping method to characterize the genetic diversity of *P. falciparum* [64]. Although it allows for a rapid evaluation of samples on a large scale with sufficient specificity and discriminatory power, it is important to realize that it requires large sample volumes. The polymorphic region is amplified first by PCR, and then one or more restriction enzymes are used with the PCR products being used as templates. The digested fragments can be analyzed using polyacrylamide gel electrophoresis. This may result in a specific banding pattern, which can distinguish different alleles within the genotyped markers [65].

## 6. Interpretation and Technical Considerations

Although primers targeting an allele-specific family sequence in *msp-1*, *msp-2*, and *glurp* are highly specific, PCR products are observed only when *P. falciparum* DNA is present [20]. Only one band may be seen on a gel if a single clone of parasite is expected. Typically, observing multiple bands in a given PCR product is interpreted as an infection with multiple clones or genotypes of the parasite [16]. However, it is important to note that two bands with the same molecular size may not necessarily represent the same allele as two different alleles may have the same molecular size but differ at the sequence level [20]. In addition, this may underestimate the allele frequency as the distinct genotype of the parasite might not be differentiated in such an analysis. However, direct sequencing of the amplified region would overcome this issue, and although using this method is the best way to differentiate between alleles with the same fragment size that differ at the sequence level, it may fail in cases of alleles carrying multiple clones.

Despite the fact that the genotyping of *P. falciparum* has been described in various studies [46,55,56], there is no consensus on the analysis and interpretation of outcomes, rendering it difficult to compare clinical trials and studies [43,44]. Although it is an arbitrary cut off, it is generally accepted that DNA fragments within 20 base pair ranges are considered one allele for *msp-1* and *msp-2*, and those within 50 base pair ranges are considered one allele for *glurp* [16,66]. This method can group alleles with a similar fragment size; however, it does not detect the presence or absence of single-point mutation that may be present across amplified regions [20]. Direct sequencing may be the gold standard to distinguish these alleles, but this method is not practical, especially in field situations [16]. Compared to regular gel electrophoresis, capillary electrophoresis can be used, which increases the resolution, and 2–3 base pair differences might be detected [25]. Discrimination between new infection and recrudescence can be complex, especially when using more than one molecular marker. In drug efficacy studies, the genotypic pattern of the parasite which appears during the time of admission is compared with the genotype of the reappearing parasite to discriminate between true recrudescence and a new infection [16]. However, although there is no consensus in how to define recrudescence and a new infection, it is generally accepted that a new infection is considered when all alleles in the parasites observed following treatment are dissimilar from those observed at the admission time for one or more loci [16]. An infection is categorized as recrudescence when at least one allele is shared at each locus in both paired samples [16]. These two definitions are important as they are mutually exclusive. The sample is either categorized as recrudescence or a new infection, but both categories are never assigned as the same time.

It should be noted that it is recommended that for molecular genotyping, blood samples must be taken right before treatment is initiated and on the first reappearance of parasitemia following the clearance of the parasite. Genotyping should be performed sequentially using three markers, *msp-1*, *msp-2*, and *glurp*, and once the infection has been defined as a new infection, the analysis should be stopped. In such a case where no evidence of a new infection is determined by the first marker, the second marker should be analyzed, and if no new infection is indicated, the third marker is used as the last marker. If the analysis of all markers excludes the possibility of a new infection, then the sample would be defined as recrudescence [16].

## 7. Conclusions

Genotyping *P. falciparum* is critically important to discriminate between a new infection and recrudescence to evaluate antimalarial drugs’ efficacy. Three key genetic markers, *msp-1*, *msp-2*, and *glurp*, are mostly used to characterize the genetic diversity of *P. falciparum*. These markers should be used sequentially until a new infection or recrudescence definition is met. Even though discriminating between these two categories can be complicated, confidence in the result can be increased by actions such as protection from further mosquito bites, using a drug that can target the liver stage, and ensuring that enough consecutive samples are analyzed. Genetic diversity and characterizing parasite populations can provide valuable information for selection processes that might be induced by vaccines, mosquito control, or environmental changes. More importantly, this information is needed for decision making and policy making regarding the use of new or established drugs.

## Figures and Tables

**Table 1:** Three genetic markers most commonly used to characterize genetic diversity of *P. falciparum*.

Gene	Expression	Chromosome	Molecular Mass	Variable Region	Allelic Family
*msp-1*	Merozoite	9	190–200 kDa	Block 2	K1, MAD20, RO33
*msp-2*	Merozoite	2	30 kDa	Block 3	FC27, 3D7
*glurp*	Sporozoite, gametocyte	10	145 kDa	RII repeat region	

**Table 2:** Family-specific primers [20] used to amplify *msp-1, msp-2*, and *glurp* genetic markers.

Genetic Marker	Primer Name	Sequence (5′ → 3′)
*msp-1* primary	M1-OF	CTAGAAGCTTTAGAAGATGCAGTATTG
	M1-OR	CTTAAATAGTATTCTAATTCAAGTGGATCA
MAD20	M1-MF	AAATGAAGGAACAAGTGGAACAGCTGTTAC
	M1-MR	ATCTGAAGGATTTGTACGTCTTGAATTACC
K1	M1-KF	AAATGAAGAAGAAATTACTACAAAAGGTGC
	M1-KR	GCTTGCATCAGCTGGAGGGCTTGCACCAGA
RO33	M1-RF	TAAAGGATGGAGCAAATACTCAAGTTGTTG
	M1-RR	CATCTGAAGGATTTGCAGCACCTGGAGATC
*msp-2* primary	M2-OF	ATGAAGGTAATTAAAACATTGTCTATTATA
	M2-OR	CTTTGTTACCATCGGTACATTCTT
3D7	M2-ICF	AGAAGTATGGCAGAAAGTAAK * CCTY ** CTACT
	M2-ICR	GATTGTAATTCGGGGGATTCAGTTTGTTCG
FC27	M2-FCF	AATACTAAGAGTGTAGGTGCAR *** ATGCTCCA
	M2-FCR	TTTTATTTGGTGCATTGCCAGAACTTGAAC
Glurp primary	G-OF	TGAATTTGAAGATGTTCACACTGAAC
	G-OR	GTGGAATTGCTTTTTCTTCAACACTAA
RII	G-NF	TGTTCACACTGAACAATTAGATTTAGATCA
	G-OR	GTGGAATTGCTTTTTCTTCAACACTAA

* K: G or T; ** Y: C or T; *** R: A or G.
